# Valedictory Address

**Published:** 1866-04

**Authors:** C. W. Spalding


### VALEDICTORY ADDRESS.

BY PROF. C. W. SPALDING.

[Delivered to the Graduating Class of the Ohio College of Dental Surgery, Feb. 21st, 1866.]

Gentlemen Graduates:—The time has arrived when you are about to leave this Institution, to engage in the active and responsible duties of professional life. Having completed the required course of study, preparatory to assuming these important duties; and having passed a thorough and searching though satisfactory examination, you now hold in your hands, as you are entitled to, the evidences of a successful pupilage; and are prepared to change your relations to your late instructors, and to take your places in society as professional men.

Speaking, as I do, to-night, in behalf of my associates, as well as myself, I extend to each of you a hearty welcome, and cordially receive you into the ranks of our professional brotherhood. The board of gentlemen who hold in trust the interests and the authority of this institution, has conferred upon each of you the title of Doctor of Dental Surgery.

This, gentlemen, is no meaningless title — conferred as a mere mark of favor or respect—but it is a well-earned passport to the confidence of the public, and *our* guarantee that you possess the qualifications necessary to the beginning of your professional career.

The term, Doctor, literally signifies one who teaches; and it was formerly applied to those only, who were so well versed in some particular art or profession, as to be qualified to teach it to others. In Law and Divinity, it still retains much of its original meaning; but in Medicine and Surgery, both general and special, the term is accepted and employed in its more modern sense, and implies one who is qualified to practice in any department of the healing art.

The conferring of this degree, therefore, invests you with all the rights and privileges which belong to any other possessor of a similar title.

In terminating, as we do with these exercises, our relations to you as teachers, the Faculty of the Institution has imposed on me the pleasing duty of addressing to you a few words of parting counsel and advice.

In terminating these *relations*, however, we do not wish you to regard—either our interest in your welfare, our earnest desire for your success in your new vocation, or our sincere wish that you may continue to pursue the course of study you have so auspiciously begun—as having, in *any* sense terminated. But, though you may disperse in various, and even opposite directions, our eyes will follow you all; and go where you will, you will always be accompanied by our best wishes for your success, and our anxious solicitude for your welfare; and while we bid you "God speed" in your journey over the laborious path of a professional life, our watchful care will ever follow you; and we shall look, with earnest yearning, for those manifestations of professional progress, which come only as the result of self-sacrificing toil. Wholly disinterested labor, however, must not be expected of any one in the daily pursuits of life. Necessity compels us to labor for ourselves, as well as for others; but fortunately, and I believe providentially, society is so organized and constituted, that men are forced to perform uses to others, whether they will or not, that they may themselves reap the highest reward of their own efforts.

The highest mark a man may set before himself, is the performance of a life of use—use to himself, and to those dependent on him; and use to his fellow-man, and thus to society. Let us, then, cheerfully co-operate with this law, so imperative in its demands upon us. It is a spark of heavenly life, infused into the condition of earthly success. It is one of those providences of God, in which the doing of good to others, is made co-incident with the performance of the duties of our daily occupation.

Nearly allied to this law, is the law of compensation. Sooner or later, man reaps the reward of all his acts. The violation of a moral or spiritual law, brings its inevitable consequences; it may be more slowly, but not less surely, than does the violation of a physical or natural law. The laws of life are universal, differing only in degree; hence the penalty of violation accords with the nature of the offense. How important is it, then, that men should always pursue the right! There is no maxim given for the guidance of man, to which he can more safely trust, than that golden one, of "doing unto others as he would that others should do to him." Make this, therefore, the rule of your professional lives. In all you do, let this maxim be your constant guide. If you are about to render any professional service, or if you have performed any professional act, ask yourselves this question, "am I acting, or have I done this in accordance with the requirements of this golden rule?" If you have not, then lose no time in retracing your steps; and, with unyielding determination, bring all up to the standard which this law exacts. In this is your safety. In this direction lies the path which will lead you to success.

In many of the operations which you will be called on to perform, different modes of procedure will present themselves for your consideration. That which your judgment will indicate as the best, may not, to you, be either the easiest or the most profitable. Let none of these considerations, however, swerve you a hair's breadth from the straight-forward path of duty. Whatever you do, do it according to the best of your ability. Let your efforts be directed, rather to improve the quality, than to increase the number of your operations. Operate well, rather than much; and do not make haste to acquire an extensive practice; progress more slow is more sure; too sudden prosperity is not the most useful. Strive, therefore, to do everything, even the smallest and the least important, in the *best possible* manner. So will you win your way to favor, with your patrons; and an ample field will soon open itself before you.

There are, also, other minor considerations, which go far in influencing persons in the choice of a dentist. Among them I may name, age, dress, deportment, the location and fittings of an office, and, quite as important as any I have mentioned, is cleanliness, absolute, scrupulous cleanliness of person and of premises. No man, who disregards this latter, can ever expect to acquire, or to retain a respectable practice. The highest attainments, and the most artistic skill, will be likely to go unrewarded, if the individual lacks this essential quality. The instruments you employ, should always be scrupulously clean, and kept well polished. A set of plain ones, kept in such condition, will be far more pleasing to your patients, than those of the most elaborate and expensive kind, kept in a slovenly or negligent manner. Your own dental organs should also serve as an example, and a type of that care and cleanliness which are so necessary for their proper preservation. Punctuality in keeping your appointments, is another important element of success; for you may be assured, that for any want of punctuality on your part, your patients will abate their own. Be always, therefore, at your post; and, if one who has tried it may advise you, do not engage in politics, nor in any pursuit not directly connected with the practice of your profession. These things will be certain to distract your attention, and will be attended with loss, in both reputation and patronage. The study and practice of dentistry, are quite sufficient to occupy your time and attention; and will leave you no leisure to devote to other topics, or to other pursuits.

Your duty, therefore, is plain. Go straight on towards the high mark of excellence which you have set before yourselves, turning neither to the right hand nor to the left. In all things be guided by principle. Discharge your duty manfully, earnestly, courageously, *conscientiously*, and all duly needful will be added unto you.

In closing the period which you have devoted entirely to study and preparation, do not imagine that the change involves the substitution of practitioner for student; the former is merely added to the latter, and if, thereafter, you reach that degree of eminence, which is in store for those who labor for its attainment, you are to do so by combining the duties of both. He who neglects study, does not even retain that which he has already acquired; while he who would keep pace with the rapid advancement which is taking place in dental art and science, must avail himself of all the helps that lie within his reach.

Cultivate, therefore, habits of study, persevering, untiring study. It is a ready means of holding converse with the learned. Who would not esteem it a great privilege to enjoy the benefit of a daily talk with some eminently scientific man? In his works, you may obtain the result of his life's labor, and research, and thought. Devote, therefore, a part of each day to the reading of such authors, and never consider the daily task accomplished, till you have had your hour of talk with your learned friends. A single hour, set apart each day, (omitting, of course, the Sabbath), would give over 300 hours in the year. Taking eight hours as a full day's study, nearly 40 full days in every year would be given to the work of improving the mind. The pleasures of social life, public and private recreations, are all proper in their places, and should be moderately engaged in. Let not the allurements of society, however, withdraw you from your purpose. Subordinate such pleasures to the more important claims which the interests of your profession have upon your time.

Let your college text books, and standard dental works, become "as familiar to you as household words." Read, also, the periodical literature of your profession, which comes to you at stated times, as a reminder and a help to you in your work; and, especially also that of medicine, and, I may add, that of the collateral sciences. In this way, gentlemen, you will lay broad and deep the foundation of your professional edifice. With a sure foundation to rest upon, the superstructure will be easily and rapidly built up.

In this connection, I cannot too strongly urge upon you the study of special cases. Whenever, in your practice, a case presents itself, which you do not fully comprehend, let your study of all its peculiarities be so completely exhaustive of the subject, that you leave nothing unlearned, concerning it, which can be obtained from books. The knowledge acquired by this method of study, becomes more indelibly fixed in the mind, than that obtained by more general reading; and having mastered this case, you will always confidently attempt the management of any similar one that may fall into your hands.

There is another custom which I would recommend to you as deserving of your attention, and that is, the practice of committing your thoughts to paper. Thoughts are evanescent things, and, unless they make an unusual impression on the mind, are apt to pass away and be forgotten. If, however, they are once written, if we once ultimate them in characters at our fingers ends, they acquire a lodgment in our memories, which they do not acquire in any other way.

If we merely contemplate and reflect upon an act, it never becomes so real as it does when the act itself is performed. So our thoughts acquire a fixity by being spoken, and a still greater one by being written, which they never would have done, had they remained in the mind merely as thoughts. I know an individual who has frequently kept paper and pencil lying upon his instrument table, and, at short intervals occuring in his operations, would turn to his paper, and there record a passing thought or two, which were afterwards valuable to him, and which would otherwise have passed away, and could not, probably, have been recalled. It is, therefore, for your own advantage that this practice is recommended; yet, in the future, we may hope that it will become advantageous to others, that you should have cultivated the habit of preserving your thoughts in this permanent and convenient form.

A prominent feature which marks the progressive spirit of the age, is the division of medicine into specialties. Indeed this condition may now be said to have become an established necessity. This division of labor has long been recognized as necessary, in all the departments of industrial life; and, having established itself in this basal stratum of society, it is now claiming increased attention at the hands of professional men. In no pursuit can it be more advantageously extended than in that of medicine. The treatment of diseases of the eye and ear, serves as an example, as well as those of the teeth. The dividing the treatment of the diseases of these organs into specialties, each having its separate set of practitioners, who devote their thoughts and their energies to the development of their particular branch, and many of whom have pursued a course of study designed to fit them for their peculiar duties, has resulted in bringing to light the true character of many diseases, the causes and nature of which were not previously at all, or but very imperfectly, understood.

The complex, and far too manifold duties of the old time physician, has so far given place to this separation into specialties. This work must go on; and it will more and more increase, in proportion as the practitioner in each department shall, by a careful and thorough study and investigation of his particular allotment, exhibit to the world, and to the medical fraternity, a higher degree of professional skill than has heretofore been attainable by the general practitioner.

And now, gentlemen, a few words touching the commencement of your professional career: This is *your* commencement; from this time you are to date the beginning of your professional lives. In entering upon the untried duties of your new vocation, you must expect to encounter many difficulties, and to meet with many discouraging circumstances. Do not allow these, however, to shake your purpose, or for a single moment to turn you aside Such is the common lot of all, and such experiences are often necessary to develope the hidden energies that lie dormant within the man.

In introducing yourselves into new localities, you must resort to some means of informing the public that your services can be obtained. An advertisement, announcing your name, occupation, and location; and, subsequently, a standing newspaper card, are all proper and right, as a means of attracting attention where you are unknown. Avoid, however, all ostentatious display, either in signs about your door-way, or in newspaper advertising. Neither offend your fellow-practitioners, nor debase yourselves, by claiming to possess *peculiar* merits, which entitle you, above others, to the confidence and patronage of the public. Let your works give you publicity; and rise, rather upon your own merits, than by a resort to what are called "the tricks of trade."

If you pursue the course I have here imperfectly sketched, you will become an ornament to your profession, and an honor to the institution, to whose interests, I am sure, you are far from being indifferent. If you would realize the hopes which these remarks are intended to excite, you must steadily exercise those trying, but sterling virtues, patience and perseverance. You must work your way, by slow, but certain steps. There is no royal road to eminence, in any walk of life; and he who aspires to reach the summit of professional attainment, must first "learn to labor and to wait."

These, then, are our parting words! This is our final counsel and advice! That you will heed the one and accept the other, we have the highest assurances, drawn from our intercourse with you in the months that are past. As you do this, so will you reflect credit upon yourselves, and on your chosen profession, and fulfill the expectations and the hopes of your ALMA MATER.

